# The Barrier–Microbiota–Inflammation Axis in Colorectal Cancer: Mechanisms and Emerging Diagnostic & Therapeutic Strategies

**DOI:** 10.3390/cancers18040576

**Published:** 2026-02-10

**Authors:** Xuanchi Dong, Ji Yang, Langyu He, Huan Fang, Lei Wang, Jingjing Zhu, Jie Xu, Kedong Song, Zhiqiang Xuan

**Affiliations:** 1 Department of Gastrointestinal Surgery, Central Hospital of Dalian University of Technology (Dalian Municipal Central Hospital), Dalian 116021, China; dxc336756@163.com (X.D.); 18002454327@163.com (J.Y.); 18040015622@163.com (L.W.); 2 Cancer Hospital of Dalian University of Technology, State Key Laboratory of Fine Chemicals, Dalian R&D Center for Stem Cell and Tissue Engineering, Dalian University of Technology, Dalian 116024, China; 2634073351@mail.dlut.edu.cn (L.H.); hfang@mail.dlut.edu.cn (H.F.); jjzhu@mail.dlut.edu.cn (J.Z.); 3 Rehabilitation College, Affiliated Rehabilitation Hospital, Jiangxi Medical College, Nanchang University, Nanchang 330003, China

**Keywords:** colorectal cancer, intestinal barrier, gut microbiota, inflammation, early diagnosis

## Abstract

Colorectal cancer (CRC) is a leading cause of global cancer incidence and mortality, with rising prevalence among younger individuals. Accumulating evidence reveals a critical pathological axis linking intestinal barrier disruption, gut microbial dysbiosis, and chronic inflammation, collectively driving CRC initiation. Early colorectal lesions exhibit microbial shifts—reduced α-diversity with clear β-diversity separation, enrichment of oral-origin/pathobiont taxa and depletion of butyrate producers—alongside impaired mucus and tight junction integrity. Concurrent chemical barrier drifts, with decreased short-chain fatty acids and increased secondary bile acids, enhance epithelial stress and vulnerability. The resultant permeability facilitates the translocation of microbial products, triggering inflammation and tumorigenesis. This paper focuses on a review of the relationship between gut microbiota, intestinal barrier, inflammatory signaling pathways, and tumor initiation, sorting out its potential role in developing, diagnosing, and treating CRC. Collectively, this cascade axis offers theoretical and empirical support for the early pathological detection and intervention of CRC, indicating promising directions for future precision screening and stratified management strategies.

## Simple Summary

Colorectal cancer is one of the most common malignancies worldwide, and its development is closely linked to intestinal barrier dysfunction, dysbiosis of the gut microbiota, and chronic inflammation. Increasing evidence suggests that these three factors do not act independently but instead form a tightly interconnected barrier–microbiota–inflammation axis that drives tumor initiation, progression, and therapeutic resistance. In this review, we summarize current advances in understanding how epithelial barrier disruption alters microbial composition, how microbiota-derived signals shape inflammatory and immune responses—particularly Th17 and regulatory T cells—and how these processes collectively influence the colorectal tumor microenvironment. We further discuss the clinical implications of targeting this axis for early diagnosis, prognosis, and the development of novel therapeutic strategies. A better understanding of this integrated axis may provide new opportunities for precision medicine in colorectal cancer.

## 1. Introduction

Colorectal cancer (CRC) remains one of the most frequently diagnosed cancers and a leading cause of cancer-related mortality worldwide. According to the most recent GLOBOCAN 2024 update, an estimated two million new cases and one million deaths occurred in 2022 [1]. There is a concerning rise in early-onset CRC across multiple regions, with studies reporting sustained increases in incidence among individuals under fifty [2,3]. Concurrently, screening participation and adherence remain inadequate in many middle-income countries, including China, where low coverage and poor follow-up after invitation are commonly reported [4]. These limitations highlight an urgent need for more acceptable and biologically informed strategies that can complement or triage colonoscopy.

Accumulating evidence indicates that intestinal barrier impairment and gut microbiota remodeling are detectable at the adenoma and early cancer stages rather than solely in advanced carcinomas [5,6]. Large-scale metagenomic analyses have consistently shown reproducible diversity shifts and enrichment of CRC-associated bacteria, such as *Fusobacterium nucleatum* (*F. nucleatum*) and pks-positive *Escherichia coli* (*E. coli*) [7]. Host-side changes include structural barrier defects—thinning of the mucus layer, loss of goblet-cell function, and downregulation of tight-junction proteins like zonula occludens-1 (ZO-1) and occludin—which collectively increase intestinal permeability and promote microbial translocation [8,9]. Shifts in the chemical barrier, such as elevated microbiota-derived secondary bile acids, have been associated with the suppression of antitumor immunity, epithelial stress, and low-grade inflammation [10,11]. In contrast, short-chain fatty acids (SCFAs), particularly butyrate, are generally considered barrier-protective metabolites that support epithelial homeostasis and immune regulation [12]. These alterations activate pro-tumor inflammatory signaling, including lipopolysaccharide (LPS)-driven toll-like receptor 4 (TLR4)/myeloid differentiation primary response 88 (MyD88)/nuclear factor-κB (NF-κB) (TLR4–MyD88–NF-κB) and *Fusobacterium* adhesins-mediated Wnt–β-catenin pathways [9,13]. This mechanistic axis not only offers novel biomarkers in stool and blood but also informs targeted strategies aimed at restoring barrier integrity and microbial balance [7].

**Figure 1:**
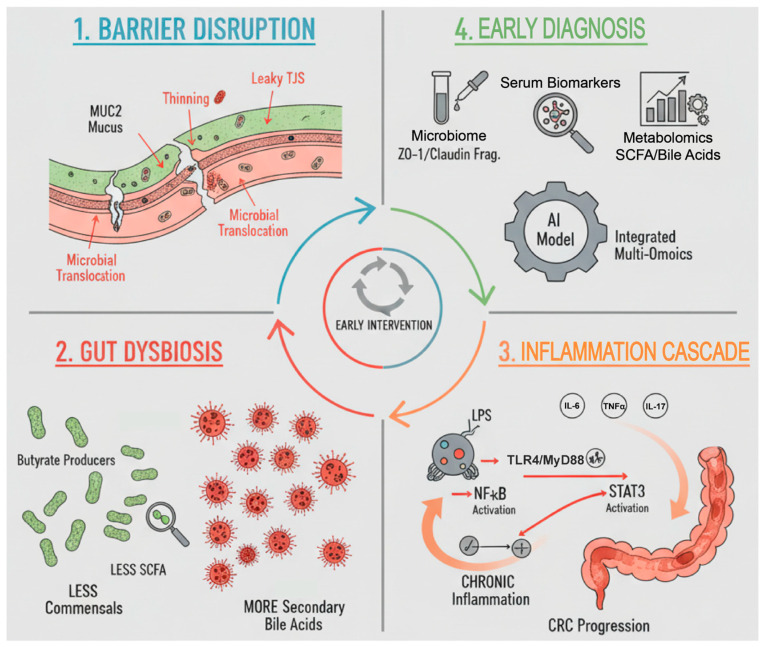
The CRC Gut Axis: Barrier Disruption, Gut Dysbiosis & Inflammation Cascade. This diagram illustrates the vicious cycle wherein intestinal barrier compromise drives gut dysbiosis, which in turn fuels chronic inflammation, thereby generating a pro-carcinogenic microenvironment. Current intervention strategies focus on these interconnected processes; however, clinical translation remains challenging due to the issues of standardization, system bias, and the requisite for extensive cohort validation. Abbreviations: CRC, colorectal cancer; LPS, lipopolysaccharide; SCFAs, short-chain fatty acids; NF-κB, nuclear factor kappa B; STAT3, signal transducer and activator of transcription 3; TLR4, Toll-like receptor 4; MyD88, myeloid differentiation primary response 88.

While current screening primarily relies on colonoscopy and fecal immunochemical testing (FIT), meta-analytic estimates confirm that FIT has limited sensitivity for advanced adenomas and variable performance in programmatic settings [14]. Recent work demonstrates that combining FIT with blood/stool-based biomarkers and age can improve diagnostic accuracy and resource allocation [15]. In this context, this review focuses on the earliest window of tumorigenesis—adenoma and early colorectal cancer—and integrates microbial alterations with mechanical and chemical barrier disruptions, mapping these changes to inflammation nodes that are actionable for diagnosis and intervention. We first address barrier disruption, then microbiome changes with sample-type and stage stratification, followed by the inflammatory cascade and its feedback loops, and finally discuss diagnostic markers and interventions aligned to each node. Importantly, within this framework, inflammation is not treated as a downstream consequence alone, but as a core and dynamic component that both results from and actively reinforces intestinal barrier dysfunction and microbial dysbiosis. To integrate these interrelated processes, we propose a conceptual framework in which intestinal barrier disruption, gut microbiota dysbiosis, and chronic inflammation form a self-reinforcing axis that drives colorectal carcinogenesis, as illustrated in Figure 1.

This narrative review is based on literature retrieved from PubMed, Web of Science, and Scopus, using combinations of keywords related to colorectal cancer, intestinal barrier function, gut microbiota, and inflammation. Priority was given to recent and representative studies with mechanistic relevance or translational significance. The aim was to integrate key findings rather than to provide a systematic or exhaustive synthesis.

## 2. Intestinal Barrier Disruption and Colorectal Cancer

### 2.1. Physiological Functions and Structural Characteristics of the Intestinal Barrier

The intestinal barrier serves as the first interface between the host and the external environment. Beyond mere physical separation, it is a multifunctional system comprising the epithelial cell layer, the mucus layer, and the mucosal immune system, which together perform diverse and dynamic regulatory roles. A key component is the epithelial tight junctions (TJs), which govern selective paracellular permeability. Occludin, a transmembrane protein, acts primarily as a sealing element, while the junctional protein ZO-1 stabilizes the TJs and links them to the actin cytoskeleton to mediate stress responses [16]. Deficiency in either occludin or ZO-1 alone may not critically disrupt barrier integrity under homeostatic conditions; however, their combined loss under inflammatory or neoplastic conditions leads to severe barrier failure, creating a hazardous vulnerability [17]. The mucus layer, primarily composed of the gel-forming protein mucin2 (MUC2) secreted by goblet cells, prevents direct contact between microbes and epithelial cells. Reduced or aberrantly glycosylated MUC2 is associated with enhanced microbial penetration, inflammation and carcinogenesis [18,19]. Similarly, the mucosal immune system, through mechanisms such as polymeric immunoglobulin receptor (pIgR)-mediated transport of polyclonal immunoglobulin A (IgA), helps manage microbial antigens. Secretory IgA (sIgA) represents a critical immune barrier component that maintains microbial homeostasis by limiting bacterial adherence to the epithelium and shaping microbial spatial organization through immune exclusion [20]. Dysregulation of pIgR-mediated IgA transcytosis or reduced sIgA coating capacity compromises this control, allowing excessive microbial–epithelial contact and promoting dysbiosis-associated inflammation [21]. In the context of CRC, impaired sIgA function has been associated with altered microbial composition and increased mucosal permeability, thereby reinforcing the barrier–microbiota–inflammation axis [22]. Impaired IgA transport can lead to pathogenic dysbiosis, heightened inflammation, and increased cancer risk [23].

The barrier possesses inherent renewal capacity via epithelial stem cells [24]. However, chronic pathological environmental stimuli can compromise its integrity, increasing permeability and activating procarcinogenic signaling pathways. The integrity of the intestinal barrier is maintained by a dynamic process of renewal and regulation, central to host homeostasis and cancer prevention [25].

In CRC-prone contexts, mechanical barrier failure becomes quantifiable, featuring reduced junctional continuity of ZO-1/occludin with cytoplasmic relocalization, thinning of the inner MUC2 layer, and glycan abnormalities [26,27]. These structural defects correlate with elevated biomarkers of permeability—such as serum LPS, anti-LPS/flagellin antibodies, diamine oxidase (DAO), and fecal/serum zonulin—which can be measured alongside TJ protein fragments in translational studies [28,29]. Mechanistically, signaling pathways such as TLR4→MyD88→NF-κB/myosin light chain kinase (MLCK) promote TJ disassembly and paracellular leak, while disruption of E-cadherin further impairs adherens junctions, facilitating microbial translocation [30,31,32]. This LPS–TLR4–MyD88–NF-κB pathway represents a central inflammatory signaling axis within the barrier–microbiota–inflammation network and will not be re-described in detail in subsequent sections.

### 2.2. Mechanisms Linking Intestinal Barrier Disruption to CRC Development

**Figure 2:**
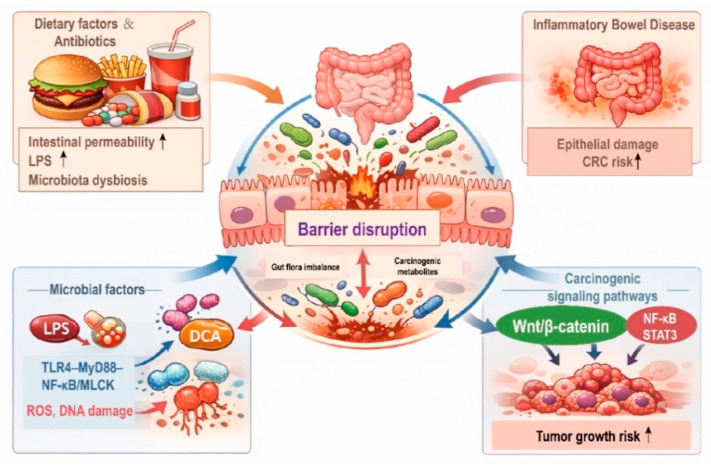
Key mechanisms linking intestinal barrier disruption to colorectal cancer development. Disruption of the intestinal barrier leads to increased intestinal permeability, microbial translocation, and activation of inflammatory pathways. Microbial factors, such as LPS, drive inflammation through TLR4–NF-κB signaling, contributing to carcinogenic signaling via pathways like Wnt/β-catenin. This cascade ultimately increases the risk of CRC development.

The compromise of the intestinal barrier acts as both a contributor and a consequence of early CRC-associated dysbiosis, establishing a feed-forward loop in which microbial products and metabolites progressively erode junctional integrity and mucus structure (Figure 2) [33,34]. Recent research suggests a high-fat diet (HFD) can directly alter the intestinal epithelial structure by reshaping the microbiota, leading to dysbiosis and the accumulation of carcinogenic metabolites [35]. Mouse models confirm that HFD or high sucrose diets downregulate tight junction protein expression, increase intestinal permeability, dramatically increase LPS levels, and promote the development of large and more numerous colorectal tumors [36]. Beyond diet, potent antibiotics can severely perturb microbial homeostasis, favoring the expansion of pathobionts, thinning the mucus layer, and thereby enhancing bacterial adhesion, transcytosis, and invasion. Furthermore, inflammatory bowel disease (IBD) induces chronic inflammation that extensively damages mucosal architecture and the intestinal barrier, which significantly increases CRC risk [37]. Clinical epidemiologic meta-analyses suggest that IBD patients face a substantially increased relative risk of CRC after a disease duration of 10 years or more [38]. Beyond serving as a permissive inflammatory environment, chronic barrier damage in these contexts also primes epithelial cells for intracellular oncogenic signaling, thereby lowering the threshold for malignant transformation.

The breach of the intestinal barrier allows luminal contents to infiltrate the lamina propria, where they directly interact with intestinal stem cells or immune cells to initiate pro-carcinogenic processes. For example, in vitro studies where LPS triggers the secretion of pro-inflammatory cytokines, downregulates tight junction proteins, promotes epithelial cell migration, and induces epithelial-to-mesenchymal transition (EMT). Importantly, these effects are mediated through intracellular activation of TLR4-dependent NF-κB and STAT3 signaling pathways, directly linking barrier-derived stimuli to epithelial cell reprogramming [39,40]. Similarly, deoxycholic acid (DCA) exhibits direct cytotoxicity toward colonic epithelial cells, induces reactive oxygen species (ROS) production, causes deoxyribonucleic acid (DNA) damage, and increases mutation rates in key genes such as p53 [41]. At the intracellular level, DCA-induced oxidative stress and DNA damage promote genomic instability and facilitate mutations in key tumor suppressor genes, reinforcing its role as a direct epithelial carcinogen rather than a purely luminal toxin [42]. Both molecules also enhance the permeation and accumulation of other carcinogens by upregulating permeability-associated channels like pIgR, thereby establishing a pathological cycle of chronic leakage, persistent stimulation, and genomic instability.

Following barrier disruption, multiple epithelial cell–intrinsic oncogenic signaling pathways are activated, forming a complex molecular network that drives carcinogenesis. These pathways represent core intracellular mechanisms of CRC initiation, indicating that barrier failure actively engages epithelial oncogenic programs rather than acting solely through secondary inflammation. For example, the Wnt/β-catenin signaling pathway, a well-established mediator of CRC, is coactivated by LPS and DCA, leading to the nuclear translocation of β-catenin and the upregulation of oncogenes such as Myc and cyclin D1 [43]. Concurrently, inflammatory signaling via NF-κB and signal transducer and activator of transcription 3 (STAT3) is rapidly engaged through the TLR4–LPS signaling pathway, promoting anti-apoptotic effects, proliferation, and immune suppression—all critical for tumor progression [44]. Significant crosstalk exists among these pathways; NF-κB activity enhances STAT3 expression, while stabilized β-catenin signaling promotes to form a malignant regulatory network characterized by inflammation-induced proliferation and immunosuppression [45]. Collectively, these insights reposition the intestinal barrier from a passive victim to an active upstream initiator of CRC. Compromise of barrier integrity enables molecular permeation and triggers powerful carcinogenic amplification cascades. Consequently, therapeutic strategies aimed at preserving or restoring barrier function could form the cornerstones of primary CRC prevention.

Specific microbial factors directly contribute to barrier lesions. LPS from blooms of Proteobacteria activates the TLR4–MyD88–NF-κB/MLCK axis, rapidly opening TJs and weakening fence function [30]. Enterotoxigenic *Bacteroides fragilis* (*BF*) secretes a toxin (BFT) that cleaves E-cadherin within minutes and loosens adherens junctions [46,47]. Mucin-degrading taxa such as *Ruminococcus gnavus* erode the inner MUC2 layer, bringing microbes closer to the epithelium [48]. Conversely, the loss of butyrate producers blunts G protein-coupled receptor (GPR)43/GPR109A- and histone deacetylase (HDAC)-dependent programs, which normally sustain the expression of TJ/MUC2 [49,50]. Simultaneously, secondary bile acids (e.g., DCA, lithocholic acid (LCA)) increase membrane permeability, induce ROS, and perturb farnesoid X receptor (FXR)/Takeda G protein-coupled receptor 5 (TGR5) bile acid receptor signaling, thereby reinforcing barrier leakiness and epithelial stress [51,52].

## 3. Gut Dysbiosis in the Onset and Progression of Colorectal Cancer

### 3.1. Association Between Gut Microbial Homeostasis and Colorectal Cancer

Across discovery and validation cohorts, patients with early-stage CRC and advanced adenomas consistently exhibit a remodeled gut microbiome, characterized by a 20–30% reduction in α-diversity, consistent β-diversity separation, and co-abundance networks that shift toward a *Fusobacterium*-centric structure while losing short-chain fatty acid (SCFA)-producing modules [53,54]. Both mucosal and fecal samples show enrichment of oral-origin taxa (e.g., *F. nucleatum*, *Parvimonas micra* (*P. micra*), enterotoxigenic *Bacteroides fragilis* (ETBF)) and depletion of butyrate producers (e.g., *Faecalibacterium prausnitzii* (*F. prausnitzii*), *Roseburia*, *Eubacterium rectale (E. rectale*), *Ruminococcus* spp.) [55]. Metagenomic analyses revealed decreased microbial capacity for SCFA biosynthesis, with concurrent enrichment in pathways involved in bile-acid deconjugation, 7α-dehydroxylation, LPS biosynthesis, and oxidative-stress, and improved disease classification by strain-level/single nucleotide variant (SNV) features [53]. In addition to these taxa, Streptococcus gallolyticus has also been repeatedly associated with colorectal cancer and implicated in tumorigenesis through inflammation-related and host–microbe interaction mechanisms (summarized in Table 1).

In a healthy state, the gut microbiota—comprising over a thousand microbial species—forms a dynamic and interactive symbiotic network dominated by Firmicutes and Bacteroidetes, with smaller proportions of Proteobacteria and Actinobacteria, which are collectively responsible for maintaining intestinal barrier function, metabolic homeostasis, and immune tolerance [59]. Notably, butyrate-producing bacteria such as *F. prausnitzii* and *Roseburia* spp., play a particularly vital role by modulating host inflammation, promoting epithelial repair, and serving as keystone taxa within gut ecology [60]. In contrast, CRC is marked by a decline in microbial α-diversity, reflecting diminished ecological resilience, and an expansion of procarcinogenic microbes such as *F. nucleatum*, *Bacteroides fragilis* (*B. fragilis*), and *P. micra* [61]. These compositional shifts are typically accompanied by profound functional alterations: fall in SCFA levels, dysregulation of amino acid metabolism, and abnormal transformation of bile acids. These metabolic disturbances not only disrupt epithelial homeostasis but may also accelerate the formation of a protumor microenvironment, partly through immune dysregulation such as increased IL-17-producing T helper (Th17) and decreased Regulatory T (Treg) activity [62].

A 2024 international consortium that aggregated data from 10 CRC cohorts reported an approximately 30% reduction in microbial α-diversity, distinct separation of β-diversity between CRC patients and control cohorts, and the enrichment of roughly 250 disease-associated genomes. The study also identified over 4300 SNVs significantly correlated with CRC status [63]. A multidimensional diagnostic model incorporating microbial and genomic features achieved an area under the concentration–time curve (AUC) exceeding 0.82 in training and 0.80 validation, indicating strong potential for non-invasive early detection [64]. Regional studies further substantiate these claims. For example, an Indonesian intestinal mucosal sampling study found a significant β-diversity difference (*p* < 0.01) between CRC patients and healthy controls at the genus and species level, with tumor tissues enriched in *F. nucleatum* and *Enterococcus faecalis* (*E. faecalis*), in contrast to the predominance of *F. prausnitzii* in non-tumor tissues [65]. Similarly, 16S ribosomal RNA (rRNA)-based stool studies from Turkey and Iraq reported increased abundances of *Bacteroides* and *Actinomyces* in CRC patients, alongside decreased levels of beneficial symbionts such as *Ruminococcus*, *Roseburia*, and *Prevotella* [66,67].

Notably, the composition and baseline abundance of CRC-associated microbial taxa exhibit substantial geographic and population-level variability [68]. Large-scale cohort studies have demonstrated that the prevalence of organisms such as Fusobacterium nucleatum differs markedly across regions, dietary patterns, and ethnic backgrounds [61,69]. This variability limits the universal applicability of fixed microbial signatures and suggests that diagnostic performance is often cohort-dependent [70,71]. Consequently, microbiota-based biomarkers should be interpreted within specific population contexts and require regional calibration and external validation before broad clinical implementation [72].

Collectively, these findings demonstrate that CRC-associated gut dysbiosis and functional imbalance are consistent across geographical, ethnic and dietary backgrounds. This suggests that microbial dysbiosis is not merely an associated mark of tumorigenesis but may play an essential role in driving the disease. With ongoing advances in multi-omic technologies and artificial intelligence, microbiota-derived signatures hold promise as predictive, non-invasive tools for early CRC detection and risk stratification, potentially extending to other poorly defined disease conditions. Emerging evidence further suggests that gut microbiota dysbiosis is differentially associated with consensus molecular subtypes (CMS) of colorectal cancer [73,74]. CMS4 tumors, characterized by mesenchymal features, stromal activation, and chronic inflammatory signaling, are frequently enriched with pro-inflammatory pathobionts such as Fusobacterium nucleatum, consistent with their immunosuppressive and invasive tumor microenvironment [75,76]. In contrast, CMS1 tumors, which are typically microsatellite instability-high and immune-reactive, exhibit distinct microbiota profiles that differ from CMS4 and may reflect alternative microbiota–immune interactions [77]. These observations indicate that dysbiosis–CMS associations are subtype-enriched rather than mutually exclusive, highlighting the bidirectional interplay between microbial composition, immune contexture, and tumor molecular phenotype.

### 3.2. Mechanisms of Microbial Dysbiosis-Induced Colorectal Cancer

Dysbiosis of the gut microbiota represents more than a phenotypic shift; it serves as a platform that mobilizes multiple oncogenic mechanisms. A key aspect of this is the expansion of specific, functionally activated “oncomicrobes”. *Fusobacterium nucleatum* is a well-characterized carcinogenic bacterium, enriched in CRC tissues where it utilizes the adhesin *Fusobacterium* adhesin A (FadA) to bind and invade epithelial cells [78]. FadA specifically targets E-cadherin on the host cell membrane, inducing conformational changes that promote the release and nuclear import of β-catenin [79]. Within the nucleus, β-catenin activates the transcription of Wnt target genes such as c-Myc and cyclin D1, thereby driving EMT and tumor cell proliferation [79]. In addition, *F. nucleatum* activates the TLR4–p-21-activated kinase 1 (PAK1) signaling pathway via its LPS, establishing a second route for enhancing β-catenin signaling and transcriptional activity. The dual activation of β-catenin through these pathways is a recognized mechanism by which *F. nucleatum* promotes the proliferation of cancerous cells [80].

Beyond *Fusobacterium*, polyketide synthase (pks)-positive *E. coli* strains are also frequently enriched in CRC. These bacteria produce colibactin, a small molecule toxin that can induce double-stranded DNA breaks and mutations in host cells. A recent prospective analysis confirmed a significantly increased mutation rate at the adenomatous polyposis coli (APC) gene: c.835-8A>G site in patients harboring pks+ *E. coli*, providing direct evidence for its genotoxic role in CRC development [81]. It is important to note that these oncogenic bacteria typically colonize and exert their effects in hosts with pre-existing vulnerabilities, such as impaired barrier function or immune suppression. Thus, *F. nucleatum* and pks+ *E. coli* should be interpreted as facilitators of carcinogenesis, acting as a “second-hit” on a susceptible host background. This host–microbiota interaction model refines our understanding of colorectal carcinogenesis and underscores the potential of targeting the microbiota for early, precision prevention.

The mechanisms linking dysbiosis to CRC extend beyond direct bacterial invasion. The loss of butyrate-producing taxa eliminates luminal butyrate signaling through GPR109A and HDAC-inhibition, pathways that normally support epithelial repair and the expression of tight-junction (e.g., ZO-1/occludin) and mucin (e.g., MUC2) genes [82,83]. Concurrently, oral pathobionts such as *F. nucleatum* exploit adhesins (e.g., FadA and fibroblast-activated protein 2 (Fap2)) to adhere to and invade colonic epithelium, perturbing junctional signaling; recent studies show that FadA can be secreted with amyloid-like properties, further enhancing virulence and epithelial engagement [84,85]. Secondary bile acids (BA, e.g., deoxycholate, lithocholate) contribute by increasing epithelial permeability and oxidative stress while disturbing BA-receptor axes (FXR/TGR5), thereby compounding barrier dysfunction [86,87]. Finally, LPS-driven TLR4-MyD88-MLCK signaling sustains a leaky state by opening tight junctions and up-regulating epithelial TLR4/CD14, locking dysbiosis to barrier failure [88,89].

Alterations in microbial metabolites constitute another major carcinogenic pathway. SCFAs, such as acetate, propionate, and butyrate, are the significant metabolites produced by commensal bacteria through dietary fiber fermentation. SCFAs exert anti-inflammatory effects, promote cancer cell apoptosis, and reinforce epithelial barrier function. In contrast, secondary bile acids (e.g., DCA, LCA), produced by some pathogenic bacteria, are pro-inflammatory and pro-proliferative, and directly damage DNA [11,81]. Meta-omics analyses revealed a significant increase in fecal DCA and LCA and a decrease in SCFAs in CRC patients; the ratio of SCFA to secondary bile acid dropped markedly from 49.6 to 20.6 [90]. Prospective studies, including prostate, lung, colorectal, and ovarian (PLCO) female cohorts, have significantly stratified CRC risk, where higher serum SCFAs mediate protection against CRC (odds ratio (OR) = 0.55, 95% confidence interval (CI): 0.31–0.98), whereas elevated DCA and conjugates with CRC significantly increase risk (OR = 2.8 to 3.4) [91,92]. In summary, the decline in SCFAs coupled with the rise in secondary bile acids forms a metabolic axis in CRC that disrupts barrier function and anti-tumor immunity while promoting chronic epithelial stress and pro-inflammatory gene expression. This evidence supports gut-derived microbial metabolites as active contributors, not passive byproducts, of colorectal carcinogenesis.

While SCFAs, particularly butyrate, are generally regarded as protective metabolites that support epithelial integrity and anti-inflammatory signaling, their biological effects are increasingly recognized as context-dependent rather than uniformly beneficial [93]. In normal colonic epithelium, SCFAs serve as a primary energy source and reinforce barrier function and immune tolerance. However, in transformed or metabolically reprogrammed epithelial cells, especially those exhibiting a glycolytic phenotype, intracellular accumulation of butyrate may exert distinct epigenetic and proliferative effects [94]. Experimental studies therefore suggest that the impact of SCFAs depends on local concentration, epithelial metabolic state, and disease stage, highlighting the need for caution when extrapolating their protective role across different phases of colorectal carcinogenesis [95].

Beyond metabolites, the impact of gut microbial taxa extends to local immune regulation, particularly the balance between Th17 and Treg cells, which is crucial for shaping the CRC tumor microenvironment and facilitating immune evasion [96]. CRC-associated dysbiosis contributes to this imbalance through defined mechanisms, including the enrichment of pro-inflammatory pathobionts that promote IL-6/IL-23-driven Th17 polarization, as well as the depletion of short-chain fatty acid-producing bacteria that normally support Treg differentiation and immune tolerance [97,98,99]. Together, these microbiota-driven alterations sustain chronic inflammatory signaling and impair antitumor immune surveillance within the colorectal tumor microenvironment. Under homeostatic conditions, a balance between Tregs and Th17 cell responses maintains immune tolerance while allowing for immune surveillance of the tumor. In CRC, this balance is disrupted, with an expansion of Th17 cells and a loss of Treg cell number and function, creating a chronic pro-tumor inflammatory state at the tumor site [100]. Systematic immune profiling links greater Th17 cell infiltration in CRC with higher recurrence and poorer survival, whereas the prognostic value of Treg infiltration appears to be context-dependent [101]. Furthermore, certain gut microbiota and their metabolites can drive the polarization of T-cells away from a Treg phenotype and toward a pro-inflammatory Th17 pathway. Metabolites that induce Treg differentiation are often depleted in CRC-associated dysbiosis, disrupting networks that induce immunosuppression. Clinical observations note a paradoxical increase in Th17 frequency after curative chemotherapy, and it is hypothesized that Th17 may contribute to immune exhaustion by recruiting immunosuppressive myeloid-derived suppressor cells and sustaining chronic STAT3 and NF-κB pathway activation [102].

In summary, dysbiosis-induced perturbations in the Th17/Treg balance foster a perpetual inflammatory state that benefits the tumor. This allows CRC to exploit a mechanism of immune evasion underpinned by microbiota and their metabolites. Consequently, strategies to counteract these microbiota-driven immunological shifts, such as probiotics and fecal microbiota transplant (FMT), are emerging as potential adjuncts to conventional immunotherapy in CRC.

## 4. Chronic Intestinal Inflammation and Inflammatory Cascade Mechanisms in Colorectal Cancer

### 4.1. Role of the Inflammatory Cascade in CRC Pathogenesis

Chronic intestinal inflammation is a well-established independent risk factor for CRC, substantially elevating cancer risk in patients with IBD, including ulcerative colitis (UC) and Crohn’s disease (CD). Epidemiologic studies have shown that patients with UC and CD face an approximately 2.4-fold increased relative risk (RR) of CRC, which escalates with longer disease duration and a greater extent of lesions [103]. This risk can be amplified to 5–10 times that of the general population in specific high-risk subgroups, such as patients with disease lasting over 8 years, those with co-morbid primary sclerosing cholangitis, or individuals with a positive family history of CRC [104]. Consequently, endoscopic surveillance is recommended in clinical guidelines for these groups. While earlier sections focus on how intestinal barrier disruption and microbial dysbiosis initiate inflammatory signaling, this section emphasizes the reciprocal process by which sustained intestinal inflammation actively remodels gut microbial composition. Chronic inflammatory signaling alters the intestinal microenvironment, thereby imposing selective pressures that reshape microbial ecology and reinforce dysbiosis.

The molecular mechanisms linking inflammation to CRC primarily involve canonical signaling pathways, predominantly NF-κB, STAT3, and the interleukin (IL)-16/IL-17 axis. Notably, much of the strongest causal evidence for inflammation-driven tumor initiation comes from colitis-associated models and IBD settings, whereas in sporadic CRC, these pathways are more often implicated as tumor-promoting programs within the local microenvironment rather than as manifestations of overt chronic colitis. Inflammatory cells within the gut release cytokines such as IL-6, tumor necrosis factor-α (TNF-α), and IL-17, which activate the STAT3 and NF-κB pathways in target cells. This activation promotes the expression of anti-apoptotic genes, proliferation-inducing factors, and angiogenic molecules, thereby converting the intestinal microenvironment from a state of inflammation to one that is pro-carcinogenic [105]. IL-17, produced by Th17 cells, γδT cells, and innate lymphoid cells, serves as a critical bridge between chronic inflammation and CRC. Its persistent activation of STAT3 signaling in epithelial cells drives hyperproliferation and the accumulation of DNA damage [106]. Azoxymethane/dextran sodium sulfate-induced rodent models show significant increases in IL-6 and IL-17F levels alongside STAT3/NF-κB pathway activation, while pharmacological inhibition of STAT3 reduces tumor progression [107]. Mechanistic studies further reveal that inflammation reprograms not only epithelial cells but also immune cells, such as tumor-associated macrophages (TAMs) and dendritic cells, toward tumor-promoting phenotypes, thereby amplifying the cytokine cascades. The coordinated activation of STAT3 and NF-κB thus constitutes a central signaling hub that drives CRC pathogenesis, particularly in the context of IBD [23].

### 4.2. Initiation of the Inflammatory Cascade via Barrier Dysfunction and Gut Dysbiosis

Intestinal barrier dysfunction and gut microbiota dysbiosis act synergistically to initiate the inflammatory cascade, a relationship robustly validated in animal models. However, it should be noted that many experimental systems that demonstrate a strong inflammation-driven initiation component are colitis-associated or inflammation-amplified models, whereas in early sporadic CRC, the morphological evidence of overt inflammation is often more limited, and inflammatory involvement may be low-grade or locally confined. Under physiological conditions, tight junction proteins and the mucosal layer prevent bacterial components from translocating across the intestinal epithelia. When the intestinal barrier is compromised, microbial products and metabolites gain access to the lamina propria [9]. There, they are recognized by host pattern recognition receptors, triggering downstream signaling cascades, such as those involving MyD88, NF-κB, and mitogen-activated protein kinases (MAPKs) that initiate robust cytokine production [108].

Although chronic inflammation can arise from prolonged exposure to dietary factors or dysbiosis, direct evidence from recent studies demonstrated that the transplanting fecal microbiota from CRC patients into germ-free and IL-10^−^/^−^ mice enhanced gut permeability, activated TLR4/MyD88 and NOD-like receptor thermal protein domain associated protein 3 (NLRP3) inflammatory pathways, and increased tumor burden [108,109]. Importantly, different bacteria exhibit distinct carcinogenic potentials despite potentially similar capacities to induce inflammation. For example, colonization with *E. coli* NC101 significantly increases more colorectal tumors than *E. faecalis*, emphasizing the specificity of microbial genomics in cancer promotion and/or suppression [110].

A critical feedback loop perpetuates this process: NF-kB, activated by LPS, not only exacerbates intestinal barrier dysfunction but also sustains persistent inflammation and dysbiosis. Inflammatory signaling profoundly alters the gut ecological niche by increasing epithelial oxygenation, nitrate availability, and antimicrobial peptide release, which preferentially supports the expansion of facultative anaerobic bacteria such as Proteobacteria while suppressing obligate anaerobic commensals. In parallel, inflammation-driven changes in bile acid composition and mucin structure further disrupt microbial metabolic balance, promoting the persistence of pro-inflammatory pathobionts [111,112,113]. Importantly, current evidence suggests that gut microbiota alterations in this cycle function both as a contributor of barrier dysfunction and inflammation, rather than as a unidirectional initiating factor [70]. This creates a self-reinforcing cycle wherein barrier damage, microbial translocation, and inflammatory amplification are continuously maintained [114,115]. These mechanistic insights reveal promising intervention points. Blocking TLR/NLR signaling, modulating the microbiota with probiotics, or using drugs to restore barrier integrity represent viable strategies to interrupt early cascade events for CRC prevention [116]. Clinically, biomarkers of barrier integrity—such as elevated anti-flagellin antibodies, serum zonulin or DAO levels, and a metabolite profile characterized by low SCFAs and high deoxycholate—correlate with the activation of TLR4/MyD88/NLRP3 pathways and associate with greater tumor burden upon the transfer of dysbiotic microbiota into gnotobiotic or carcinogen-primed mice [97,117,118,119,120,121]. This LPS–TLR4–MyD88–NF-κB axis therefore represents a central inflammatory signaling node within the barrier–microbiota–inflammation network and will not be re-described in detail in subsequent sections.

Collectively, these findings underscore inflammation as a core component of the barrier–microbiota–inflammation axis, dynamically linking epithelial dysfunction and microbial dysbiosis to sustained tumor-promoting conditions in colorectal cancer.

## 5. Advances in Research Targeting CRC Early Diagnosis and Intervention

### 5.1. Biomarkers for Early Diagnosis and Prediction

CRC screening is evolving from traditional morphological methods toward molecular classification and biomarker-based strategies. A major focus is the development of highly specific and sensitive indicators derived from non-invasive samples for precision diagnostics. Compromise of the intestinal barrier serves as an early diagnosis signal, as degradation products of its structural proteins can be detected in serum. For instance, tight junction proteins such as ZO-1 and Claudin-3 are markedly reduced in early lesions, and ZO-1 fragments measurable by enzyme linked immunosorbent assay (ELISA) in serum correlate positively with intestinal permeability markers like LPS and DAO [122]. Although these barrier-derived fragments alone show limited diagnostic power (AUC ≈ 0.78), their performance improves when combined with microbes or metabolic biomarkers [122].

Microbial signatures, typically derived from CRC-associated taxa such as Fusobacterium nucleatum, Parvimonas micra, Bacteroides fragilis, and Escherichia coli, represent a promising biomarker category. Enrichment of species such as *F. nucleatum*, *B. fragilis*, and *P. micra* in early CRC enables non-invasive stool screening via quantitative polymerase chain reaction (qPCR) [123]. A systematic review in 2023 demonstrated that random forest models incorporating any four species achieved AUC values of 0.86 to 0.94, outperforming established single markers like FIT and Septin 9 (AUC > 0.74) [124]. In blood, circulating microbial DNA (cfbDNA) has emerged as a novel liquid biopsy, offering an alternative for individuals reluctant or unable to undergo colonoscopy or stool testing. Metabolomics, which measure a type of host–microbiota interaction, also presents strong diagnostic potential. One multicenter study linked eight fecal metabolites to CRC, achieving an AUC of 0.94 for discriminating CRC from healthy controls and 0.92 for distinguishing CRC from adenomas, surpassing existing fecal DNA-based products [125].

Integrating microbial signatures with metabolic profiles and serum proteins further enhance the diagnostic capability. In 2023, a model that combined gut microbiota-associated serum metabolites with early CRC tissue expression achieved an AUC of 0.87 in external validation, outperforming both FIT-DNA and Septin 9 [126]. Such multimodal models provide a realistic basis for precise, non-invasive early diagnosis and could potentially replace current CRC screening methods.

Indeed, composite panels that combine microbial signatures (e.g., *F. nucleatum*, *P. micra*, ETBF), barrier-injury markers (e.g., anti-LPS/flagellin antibodies, DAO/zonulin), and metabolites (e.g., low SCFAs, high DCA) consistently outperform single-marker assays, achieving AUCs of approximately 0.83–0.90 across cohorts and improving the triage efficacy of FIT for colonoscopy referral [127,128].

Importantly, emerging diagnostic strategies should be understood not as isolated biomarkers but as operational frameworks that integrate biological signals into actionable screening or triage pathways. As summarized in Table 2, current approaches range from single-marker assays to composite panels and multimodal algorithms that combine microbiota-derived features, barrier integrity markers, metabolites, and clinical variables. These integrated strategies consistently outperform single-marker tests and are better aligned with real-world screening needs, particularly in FIT-based population programs and early-onset CRC risk stratification.

### 5.2. Early Intervention Strategies Targeting Intestinal Barrier and Microbial Ecology

With growing validation of the “barrier–microbiota–inflammation” axis in CRC, clinical research is increasingly focused on upstream interventions that are barrier-protective and microbiota-modulatory. These strategies, which include prebiotics and dedicated barrier-strengthening agents, are anticipated to form the cornerstone of prevention in high-risk or early screened cohorts. For example, zinc supplementation has been shown to enhance the expression of tight junction proteins including ZO-1 and occludin and reduce barrier-related inflammation by inhibiting the NF-κB related pathway [134]. A small prospective intervention trial found that zinc alpha-ketoacid nutritional formulations discouraged intestinal permeability in IBD patients, as measured inversely to serum barrier injury markers, such as DAO and D-lactate (*p* < 0.05), promoting the authors to suggest a potential role in CRC risk reduction [135]. Similarly, glucagon-like peptide-2 (GLP-2) analogues like teduglutide, known for supporting mucosal repair in short bowel syndrome, have been shown to stimulate MUC2 secretion from goblet cells, sustain mucous layer intactness, and promote epithelial proliferation and barrier recovery in preclinical and ongoing human studies [136]. In human studies—most robustly in short bowel syndrome-associated intestinal failure—GLP-2 therapy (e.g., teduglutide) has demonstrated clinically meaningful improvements in intestinal function, including significant reductions in parenteral support requirements and enhanced fluid and nutrient absorption, as shown in randomized controlled trials and prospective clinical studies [137,138]. These functional benefits are accompanied by evidence of mucosal structural recovery, such as increased villus height and crypt depth, reflecting enhanced epithelial regenerative capacity. Importantly, emerging clinical biomarker studies further suggest that GLP-2 treatment can modulate barrier-related molecular programs, including the expression of tight junction-associated proteins and improve functional readouts of intestinal permeability in patients with intestinal failure [139]. In addition, longer-acting GLP-2 analogues, such as apraglutide and glepaglutide, are currently under active clinical development, aiming to provide sustained intestinal adaptation with less frequent dosing and improved patient compliance [140]. While these clinical data support GLP-2 analogues as promising agents for intestinal barrier recovery, their application in CRC settings should currently be considered adjunctive and investigational, pending dedicated clinical trials addressing CRC-specific endpoints and patient populations.

These barrier-protective agents primarily act on the host epithelium by enhancing tight junction protein expression, supporting goblet cell function, and accelerating epithelial renewal, thereby indirectly stabilizing the microbial niche and limiting dysbiosis-driven inflammation [141]. Currently, there is a scarcity of large randomized controlled trials (RCTs) demonstrating that early interventions can modify precancer precursors or epithelial dysfunctions driven by intestinal permeability. Consequently, further investigation is required before progressing to more definitive large-scale trials.

Nutritional strategies also play a key role in maintaining microbiome homeostasis. Dietary fibers act as natural prebiotics, fermented by specific butyrate-producing bacteria into SCFAs—primarily butyrate—which in turn inhibit histone deacetylase and promote tumor cell apoptosis. Mechanistically, dietary fibers act as selective substrates for beneficial commensals, particularly short-chain fatty acid-producing bacteria, leading to increased luminal SCFA levels that reinforce epithelial tight junctions, promote mucus production, and suppress low-grade inflammation [142]. A 2024 Canadian translational study transplanted fecal microbiota from CRC patients into germ-free mice and then administered various dietary fibers. This intervention significantly altered microbial community structure, increased SCFA production, and improved healing of surgical intestinal anastomoses, suggesting a potential application in optimizing gut health for oncology surgery [143].

FMT, while established in IBD, is also being explored in CRC. By restoring microbial diversity and reducing the dominance of pro-inflammatory pathobionts, microbiota-targeted interventions such as probiotics and fecal microbiota transplantation also contribute to secondary improvements in barrier integrity and immune homeostasis, highlighting the bidirectional relationship between microbial composition and epithelial function [144]. A systematic review published in 2023 with 23 identified RCTs evaluated FMT’s effects in patients undergoing CRC surgery, chemoradiotherapy, or risk polypectomy, and found that it could restore microbiota diversity, reduce pro-inflammatory taxa, and help reset immune homeostasis [145]. Although not yet a first-line treatment for CRC, FMT represents a promising platform for pre- and post-surgical interventions, with future potential lying in personalized donor matching and targeted application for high-risk individuals.

Beyond their preventive potential, microbiota-targeted approaches are increasingly explored as novel therapeutic strategies in CRC patients. Probiotics and prebiotics aim to restore microbial balance by enriching beneficial commensals and enhancing the production of anti-inflammatory metabolites, thereby indirectly modulating tumor-associated inflammation and epithelial barrier function [98]. Fecal microbiota transplantation (FMT), although still experimental in CRC, has shown promise in re-establishing microbial diversity and reshaping the intestinal microenvironment, particularly in the context of inflammation and treatment responsiveness [146]. Dietary interventions further influence these processes by altering microbial substrates and metabolic outputs, highlighting the interconnected role of diet–microbiota–host interactions in CRC progression and therapy [147].

### 5.3. Therapeutic Approaches Targeting Inflammatory Cascade in CRC Prevention

Substantial evidence implicates chronic inflammation as a key contributor of CRC development and progression, making it a compelling target for prevention and early intervention. Aspirin is one of the most well-established anti-inflammatory agents for CRC chemoprevention. It inhibits cyclooxygenase-2 (COX-2), thereby reducing prostaglandin E2 (PGE2) production and modulating downstream IL-6/NF-κB/STAT3 anti-inflammatory signaling [148]. Available longitudinal evidence suggests that dietary interventions may influence CRC risk [145]. In addition, evidence suggests that aspirin may offer a particular survival benefit for patients whose tumors harbor a PIK3CA mutation [149].

Beyond aspirin, the Janus kinase-signal transducer (JAK-STAT) signaling pathway is under investigation. In CRC, the pro-inflammatory cytokine IL-6 stimulates STAT3 via JAK2, promoting immune evasion and apoptosis resistance [150]. Preclinically, the JAK2 inhibitor SAR302503 blocks STAT3 phosphorylation, suppressing proliferation and inducing apoptosis in cancer cell lines, and shows potent tumor-suppressive effects in CRC xenograft mouse models [151]. Although clinical research on JAK inhibitors in CRC remains limited, several agents have advanced to multi-site clinical trials to assess their potential as adjuncts to chemotherapy [152].

The IL-17 signaling pathway, which links immunity and gut microbiota, presents another interesting therapeutic target. IL-17A drives Th17 cell polarization, tumor proliferation, immune suppression, and cancer-associated cachexia via JAK2/STAT3 [153]. Animal studies demonstrated that IL-17A-neutralizing antibodies can inhibit tumor-promoting inflammatory mediators by blocking STAT3 activation. IL-17-targeting monoclonal antibodies such as secukinumab and brodalumab, already approved for psoriasis, are now in phase I/II clinical trials for CRC and other solid tumors [154].

Overall, the data strongly support the continued investigation of inflammation as a central mechanism and therapeutic target in CRC. Personalized strategies that inhibit the JAK-STAT or IL-17 signaling pathways, integrated with conventional screening pathways, represent a promising avenue for precision prevention and intervention in inflammation-associated colorectal cancers.

## 6. Challenges for Clinical Translation

### 6.1. Barriers to Clinical Implementation

Despite significant advances in understanding the roles of intestinal barrier function, microbiota, and inflammation in CRC, translating these discoveries into clinically usable diagnostic and management tools faces substantial hurdles. A fundamental challenge is the lack of standardized biomarker measurements. For example, there are no universally accepted reference standards for quantifying intestinal barrier-associated proteins (e.g., ZO-1, Claudin, and pIgR) in serum. Consequently, variability in antibodies, ELISA kits, and sample preparation protocols across labs compromises the comparability of results between studies [155]. Similar issues affect microbiota-based biomarkers and metabolomics models, where geographic, dietary, and sample handling differences introduce substantial variation in fecal microbial profiles, limiting the cross-study applicability of findings even when using uniform sequencing platforms like 16S rRNA [156].

Patient heterogeneity in genetic background, tumor molecular subtype, and baseline microbiota further constrains the development of universal biomarkers. The prevalence of *F. nucleatum*, for example, varies dramatically, from <20% in some southern Chinese sub-groups to ~70% in Western populations, limiting its global utility while underscoring its potential in specific screening contexts [157]. Pre-analytical variability remains another critical obstacle; even in a rigorous multicenter prospective cohort study of fresh CRC tissues, significant differences in RNA integrity number (RIN) were observed across participating institutions, despite adherence to standardized operating procedures (SOPs) [158]. Collectively, these findings highlight the urgent need for standardized, cross-center protocols in sample collection, processing, and analysis before early diagnostic models can be reliably applied in clinical practice.

In addition to standardization challenges, microbiome-based studies are subject to methodological biases that complicate cross-study comparison and interpretation. Variability in sample type (stool versus mucosal tissue), sequencing approaches (16S rRNA versus shotgun metagenomics), bioinformatic pipelines, and reference databases can substantially influence reported microbial profiles [159,160]. Moreover, confounding factors such as diet, medication use, bowel preparation, and comorbidities are often incompletely controlled [161,162]. These limitations underscore that microbiota–CRC associations are probabilistic rather than deterministic and should be interpreted with caution, particularly when extrapolating findings across platforms or populations [163].

### 6.2. Limitations in Current Research

Beyond standardization, translational research on the barrier–microbiota–inflammation axis faces inherent methodological limitations. It should also be acknowledged that these constraints reflect inherent limitations of the present narrative review, which integrates evidence from heterogeneous in vitro, animal, and human studies rather than from uniformly designed clinical trials. As such, the conclusions drawn here are intended to be integrative and hypothesis-generating, rather than definitive or predictive at the clinical level. Many mechanistic findings are derived from animal models or small-scale human observational studies, lacking validation in large, prospective clinical studies. While animal studies consistently demonstrate the tumor-suppressive effects of SCFAs, for instance, the considerable variability in human diet, host metabolism, and gut microbiota composition has precluded a consensus on their predictive value for CRC risk or their utility in existing screening programs [164].

Moreover, many CRC biomarker studies are hampered by methodological weakness, including small sample size, short follow-up period, and overly broad patient inclusion criteria, which limit the generalizability of findings. A recent systematic review reported that while most proposed CRC biomarkers reach the discovery phase, very few undergo external validation, and nearly 90% progress no further [165]. Biomarkers are also frequently treated as secondary endpoints in clinical trials, which often prioritize statistical over clinical relevance and fail to address practical outcomes like number need to treat (NNT) or cost-effectiveness.

**Figure 3:**
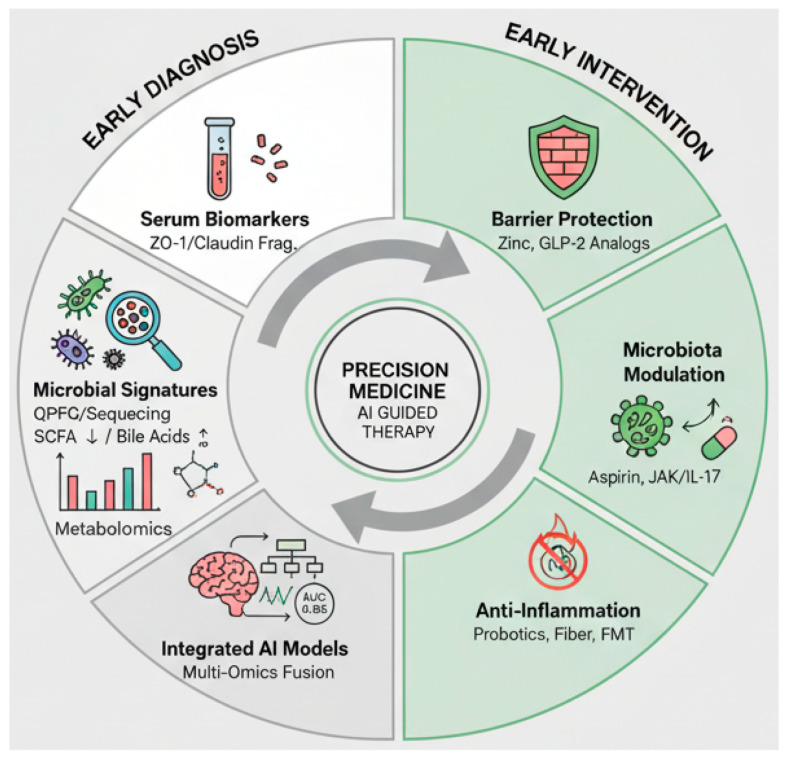
Strategies for Early Diagnosis & Intervention in CRC. This figure outlines advanced strategies for CRC. Early diagnosis integrates serum biomarkers, microbial signatures, metabolomics, and AI models for high-accuracy detection. Early intervention focuses on barrier protection, microbiota modulation, and anti-inflammation. The integration of these elements aims to advance AI-driven precision medicine, pending resolution of challenges in standardization and clinical validation. Abbreviations: FMT, fecal microbiota transplantation; SCFAs, short-chain fatty acids; AI, artificial intelligence; CRC, colorectal cancer.

Does the disconnect between basic research context and clinical contexts impede practical implementation? For instance, advanced metabolite analysis requires sophisticated, expensive equipment like precision mass spectrometers, which are impractical for widespread screening. Even more accessible techniques like qPCR exhibit considerable inter-laboratory variability (>10%) due to differences in sample handling and amplification protocols. In response, some researchers propose a “translational readiness scale” as a structured framework to systematically evaluate the feasibility and sustainability of converting laboratory findings into clinically deployable tools [166]. In summary, while the barrier–microbiota–inflammation axis holds great promise for revolutionizing early CRC detection and intervention, overcoming challenges related to standardization, methodological rigor, and clinical feasibility is essential for translating experimental discoveries into practical application. To illustrate how these insights can be integrated, Figure 3 presents a comprehensive framework that combines multi-omics data, artificial intelligence models, and targeted intervention strategies for the precision management of colorectal cancer.

## 7. Future Research Directions and Recommendations

### 7.1. Key Scientific Questions

Despite considerable progress, key scientific questions regarding the roles of the intestinal barrier function, microbiota, and inflammatory cascades in CRC remain unanswered. First, many existing studies report observational associations or validate single factors, lacking the nuanced analysis of signaling networks and causal pathways required to fully elucidate CRC pathobiology. The detailed mechanisms of microbiota-associated inflammation, such as the complex interplay between TLR, NLR, and STAT3 signaling under varying microenvironmental conditions, are still poorly defined [167]. Second, the interrelationships between structural barrier injuries, microbial metabolic shifts, and host immune modulation remain unclear, impeding the identification of specific, druggable therapeutic targets.

To address these gaps, future research should leverage advanced technologies such as organoids, single-cell omics, and spatial transcriptomics to delineate pathogenic molecular signatures driving CRC initiation and progression. Moreover, traditional single-omics approaches are insufficient to capture the complexity of multi-source biological data. Researchers should therefore adopt integrated multi-omics strategies—combining metagenomics, metabolomics, proteomics, and transcriptomics within a systems biology framework—to reconstruct a holistic landscape of CRC development and identify actionable biomarkers or intervention pathways [168].

### 7.2. Methodological and Strategic Recommendations

Bridging basic science and clinical application requires innovative methodological and strategic approaches. Firstly, there is a critical need for large, prospective cohort studies focusing on early-stage CRC or precancerous populations. These cohort studies should collect comprehensive, longitudinal multidimensional data—including gut microbiota composition, intestinal barrier integrity, metabolic transformations, multi-omics profiling, and immune markers—to build dynamic models of CRC precursor evolution. Examples like the Dutch COLOCARE and the German RaPS studies exemplify how stratified follow-up of familial and microbial profiles can clarify non-linear disease trajectories and refine personalized risk assessment [169]. Secondly, a multi-omics, systems biology-based approach should be mandated in future research. Our own work in early-onset CRC has identified key microbial and metabolic targets through multi-omic analysis [170]. Future studies should employ systems biology network modeling to dissect causal relationships within the barrier–microbiota–inflammation axis and identify intersection/panel targets at the intersection multi-pathways. Finally, developing and validating scalable, clinically translatable technologies for microbiome and metabolite detection is essential. Promising pilot studies in China and Italy are combining cell-free DNA (cfDNA) analysis, microbial qPCR, and metabolomic fingerprinting to advance broader clinical applications [171].

### 7.3. Policy and Clinical Practice Recommendations

Policy and clinical practice must align with emerging scientific knowledge. National screening guidelines should incorporate microbiome signatures and barrier function markers into risk stratification and early intervention evaluations, particularly for younger at-risk populations, to markedly improve screening precision and outcomes [172]. We further recommend establishing national funding programs dedicated to supporting the clinical translation of multi-omics discoveries, designed to foster an integrated approach encompassing mechanisms, biomarkers, tools, pathways and patient populations.

Cross-sector collaboration, clinical data collection, standardized data sharing, and streamlined regulatory pathways are also crucial. Initiatives like the European National Cancer Imaging Translational Accelerator (NCITA) demonstrate how coordinated efforts to standardize protocols, develop integrated platforms, and strengthen academia-industry partnerships can accelerate the clinical translation of biomarkers [173]. In conclusion, integrating the “barrier–microbiome–inflammation axis” into clinical practice will require co-advancements in science, technology, and policy, strategically coordinated to optimize and sustain improved strategies for early CRC screening and intervention.

## 8. Conclusions

This review has systematically analyzed the core mechanisms and translational potential of the “intestinal barrier disruption–gut dysbiosis–inflammatory cascade” axis in the early diagnosis and treatment of CRC. The axis begins with the loss of epithelial integrity, is propelled by microbial dysbiosis, and perpetuated by chronic inflammation, collectively establishing a pro-carcinogenic microenvironment. The academic significance of this framework lies in its ability to elucidate critical early disease processes in CRC pathogenesis, thereby expanding opportunities for targeted intervention and minimally invasive screening. At the molecular level, this integrative framework connects structural barrier proteins, oncogenic bacteria, and inflammatory signaling pathways, refining our understanding of tumor initiation. In diagnostic studies, combined biomarker panels—incorporating microbial signatures, metabolic profiles, and barrier integrity markers—have demonstrated superior performance (AUC ≥ 0.85) compared to single-parameter tests. In therapeutic studies, strategies such as prebiotics, GLP-2 analogues, and IL-17 antibodies are progressing toward clinical application. However, the current evidence base primarily consists of level IIb–III studies derived from small clinical cohorts, animal models, and observational data. Thus, while the proposed axis provides a comprehensive theoretical pathway from barrier dysfunction to carcinogenesis, its clinical relevance must be further substantiated through rigorous, multicenter prospective studies and well-designed interventional trials.

For the future, a precision medicine framework centered on the “barrier–microbiota–inflammation axis” offers promising applications in CRC risk assessment and management, tailored intervention, and post-operative surveillance. The integration of multi-omics data, artificial intelligence-driven predictive modeling, and microbiome-targeted therapies envisions a future of fully integrated, proactive CRC management—shifting the paradigm from traditional treatment to early interception and risk stratification. Realizing this vision will require addressing concomitant ethical and practical challenges, including safeguarding microbiota data privacy, managing the costs of expanded screening, and establishing clear regulatory guidelines. The ultimate goal is to harmonize academic rigor with considerations of patient risk and equity to ensure broad and legitimate access to these advancing strategies.

## Figures and Tables

**Table 1:** Representative evidence linking Streptococcus gallolyticus to colorectal cancer.

Study	Sample Type	Detection Method	Key Findings	Proposed Mechanism	References
Abdulamir et al. (2011)	CRC tissue/blood	PCR, culture	Higher prevalence of *Streptococcus gallolyticus* (formerly *S. bovis*) in CRC patients	Chronic inflammation and bacterial translocation contribute to carcinogenesis	[56]
Butt et al. (2017)	Serum antibodies	Serology (multiplex)	Elevated anti-*S. gallolyticus* pilus protein antibodies (Gallo2178/2179) in CRC patients	Immune-mediated association; antibody response as biomarker for CRC risk	[57]
Boleij et al. (2011)	Tumor tissue	qPCR, sequencing	Enrichment of *S. gallolyticus* DNA and antigen expression in tumor vs. normal tissues	Disruption of epithelial barrier and induction of inflammatory signaling pathways	[58]

**Table 2:** Emerging diagnostic strategies targeting the barrier–microbiota–inflammation axis in colorectal cancer.

Diagnostic Strategy	Sample Type	Core Biological Basis	Representative Markers/Models	Intended Clinical Use	Diagnostic Performance	References
Single microbial biomarker assays	Stool	CRC-associated microbial enrichment	Fusobacterium nucleatum, Peptostreptococcus anaerobius	Non-invasive CRC screening	Fn: AUC ~0.82; combined markers improve detection	[129]
Barrier integrity–related serum markers/host response	Blood	Host immune response to microbiota; epithelial barrier dysfunction	Antibodies against Fn, anti-microbial serology	Adjunct early detection	Enhanced discrimination when combined	[130]
Stool metabolic & microbial dysbiosis profiling	Stool	Dysregulated microbial metabolites & taxa	Multi-taxa microbial panels	Early adenoma & CRC detection	Microbiome AUC ~0.80–0.89	[122]
Microbiota + FIT integration	Stool (FIT + microbes)	Occult bleeding complemented by microbial signals	FIT + Fusobacterium quantification	Improved FIT screening	Sensitivity 92.3%, specificity ~93% for CRC (PMC)	[131]
Machine learning microbiome models	Stool	Gut microbial community patterns with AI	Random forest & RF microbial risk score models	Early CRC & adenoma classification	AUC ~0.82–0.90	[132]
AI-ML gut microbiome prediction tools	Stool (multi-cohort)	Species-level profiles + ML	e.g., CRCpred (XGBoost)	Population screening enhancement	AUC ~0.90–0.91	[133]

## Data Availability

The data are available from the corresponding author on reasonable request.
